# Smokeless Tobacco Use as a Risk Factor for Periodontal Disease

**DOI:** 10.3389/fpubh.2014.00195

**Published:** 2014-10-20

**Authors:** Kavitha P. Kamath, Supriya Mishra, Pradeep S. Anand

**Affiliations:** ^1^Department of Oral Pathology, People’s Dental Academy, Bhopal, India; ^2^Department of Periodontics, Maitri College of Dentistry and Research Centre, Anjora, India; ^3^Department of Dentistry, ESIC Medical College Hospital, Parippally, India

**Keywords:** tobacco, smokeless tobacco, periodontal disease, periodontal attachment loss, gingival recession

Periodontal disease is one of the leading causes of tooth loss, particularly among older individuals (1–6). Although dental plaque-associated microorganisms are the primary etiologic agents of periodontal diseases, several other factors such as local, genetic, systemic, and environmental factors play an important role in determining the susceptibility of individuals to periodontal diseases. Tobacco smoking is one of the most important environmental risk factors for periodontal diseases. Large numbers of studies have been conducted to understand the role of smoking in the etiology of periodontal diseases and the available data show that smoking is associated with increased prevalence and severity of periodontal disease, which may be due to the adverse effects of tobacco smoke on the physiology, immunology, and microbiology of the oral environment. Unlike smoking, the role of oral smokeless tobacco (SLT) in the etiology of periodontal disease has received considerably less attention.

Although traditionally, oral SLT consumption has been associated with oral malignant and potentially malignant lesions, emerging data suggest that these habits may be associated with poor periodontal health also. Besides some case reports mentioning periodontal changes associated with oral SLT habits (7), initial studies conducted in the US have shown that oral SLT habits are associated with increased incidence of gingival recession (8–11). Studies conducted in Sweden also have shown that the consumption of moist snuff, an oral SLT product, is associated with increased prevalence of gingival recession (12–14). However, some studies conducted in the US and Sweden have failed to show any association between SLT habits and periodontal changes such as gingival recession, attachment loss, or bone loss (15–18). Unlike the studies among the Swedish and US populations, studies conducted among Asian populations have shown that oral SLT habits are associated with destructive periodontal disease. Studies conducted in India have reported that oral SLT users tend to have higher scores and risk for periodontal disease (19–22). Similar results were reported among SLT users in Bangladesh and Thailand also (23, 24). A study based on National Health and Nutrition Examination Survey III data conducted in the US also showed strong association between oral SLT use and severe active periodontal disease (25).

Very few studies have reported on the pattern of periodontal destruction among oral SLT users. A study on the patterns of tooth loss among tobacco users in central India showed that mandibular tooth loss was more among oral SLT users suggesting that the deleterious effects of SLT use is manifested more in mandibular teeth (26). Studies reporting the occurrence of gingival recession among oral SLT users have reported that these occurred at sites adjacent to mucosal lesions suggesting that the recession was a result of long-term injury to the gingival tissues from the SLT product (8, 10, 13, 14). Oral SLT users in a central Indian population were shown to have an increase in prevalence and severity of recession and attachment loss at mandibular teeth, buccal surfaces, anterior teeth, and molars-the surfaces most likely to have prolonged exposure to SLT product due to retention of the SLT product at the mandibular buccal or anterior labial vestibule (21).

Oral SLT consumption in various forms is highly prevalent among all populations, particularly in the Asian countries (27–35), and a wide variety of SLT products are available worldwide (36, 37). The most common SLT products available in the US include chewing tobacco and snuff (moist and dry), and in Sweden, the most common product is snus. In Asian countries, such as India and Bangladesh, a myriad of SLT products are available such as betel quid with tobacco, zarda (prepared by boiling tobacco leaves with water and slaked lime), gutka (mixture of powdered tobacco, areca nut, slaked lime, and catechu), mawa (areca nut, tobacco, and slaked lime), and khaini (tobacco with slaked lime).

Although oral SLT habits are common among all populations, strong associations between SLT habits and destructive periodontal disease has been observed mainly among Asian populations, whereas a systematic review of studies testing the association between SLT habits and periodontal disease conducted in Sweden and the US suggested that SLT habits may not be related to periodontal diseases (38). Such contradictory observations may be due to several factors such as differences in the trends of oral SLT practices and the type of SLT products used by the respective populations.

While products such as the Swedish snus has been shown to be less harmful compared to smoking and other SLT products (39), oral SLT products available commercially in India and other Asian countries contain more than 4000 toxic ingredients, which can cause tissue injury on account of their mutagenic and carcinogenic properties (40, 41). These include alkaloids such as nicotine, tobacco-specific nitrosamines, phytosterols such as cholesterol, heterocyclic hydrocarbons, pesticides, alkali nitrites, radioactive substances, and toxic metals such as lead, cadmium, and arsenic (41). Moreover, among Asian populations, unlike smoking, which is almost exclusively confined to males, oral SLT consumption is highly prevalent among both males and females (32, 34, 42). The high prevalence of these habits may be due to a general misconception, particularly among Asian populations, that oral SLT habits are generally less harmful than smoking. While this may be true of certain products such as the Swedish snus, which is being recommended as a less harmful alternative to smoking (39, 43, 44), the products available in the Asian countries have been shown to contain large quantities of chemicals, which are toxic to the human tissues (40, 41). Moreover, such habits are culturally ingrained among these populations, which consider SLT products as relatively acceptable forms of tobacco consumption when compared to smoking, and individuals, considering it as a part of their life style, are introduced to such habits at a very young age (32, 45, 46). Lower levels of education, poor socio-economic status, and rural areas of residence have also been identified as factors associated with oral SLT consumption (31, 32, 42, 47, 48). Studies on tobacco-cessation behavior have shown that oral SLT users are less likely to attempt quitting the habit compared to smokers in spite of an awareness of the harmful effects of such habits (49, 50). On account of these variations in the products and patterns of SLT consumption, comparison between observations from Asian populations and those from European and US populations becomes difficult.

Another factor that needs to be considered while assessing the strength of association between SLT and periodontal disease is the fact that the SLT products used in Asian countries, where a stronger evidence of association is found, contains a wide variety of ingredients besides tobacco such as betel quid, areca nut, slaked lime, catechu, spices, etc. Moreover, the methods of preparation of these products also vary, which may contribute to alteration of toxicity of these ingredients. Hence, it may be difficult to identify the specific effects of tobacco on the periodontal tissues. Furthermore, the effects of these different commercial products on the periodontal tissues may also be different on account of their different chemical composition. To the best of our knowledge, no product-specific data regarding the effect of SLT on periodontal health among Indian or other Asian populations is available.

Nicotine, the principal alkaloid in tobacco, exerts a wide range of effects on the immune system and wound healing, which may play an important role in periodontal tissue destruction (51). Nicotine exposure has been shown to result in vasoconstriction (52, 53) and impaired angiogenesis (54, 55). Its effects on neutrophil function include increased shedding of adhesion molecules (56) and alteration of f-actin kinetics (57), resulting in reduced migration of neutrophils into the oral tissues (58), and inhibition of phagocytosis and oxidative killing (59, 60). Nicotine exposure also results in reduced proliferation and function of T-lymphocytes (61), decreased phagocytosis and production of pro-inflammatory cytokines and oxygen radicals by monocytes (59, 62), increased levels of tissue-destructive cytokine such as TNF-α (63), reduction in levels of antibodies to periodontal pathogens (64–67), and impaired attachment of human periodontal ligament fibroblasts (68).

Absorption of nicotine from SLT products occurs through the oral mucosa and absorption is higher from products, which have a higher pH (41, 69, 70). SLT products from different countries differ in their values of pH, total nicotine, and unionized nicotine, with products available in south-east Asian countries having higher levels of these parameters (37). On account of these differences, there are considerable limitations in drawing broad conclusions on the impact of SLT products on public health.

Nevertheless, considering the health hazards posed by oral SLT habits, particularly among Asian populations, and the high prevalence of such habits among both males and females, lack of willingness among SLT users to quit the habit is alarming, particularly since nicotine dependence and withdrawal symptoms have been shown to be higher among oral SLT users than smokers (71). In the context of such widespread use of SLT products, the implications of the effects of such habits on periodontal and oral health and on oral health-related quality of life cannot be ignored. In populations, where the tobacco consumption in the form of oral SLT products is predominant compared to smoking, the role of oral SLT products on periodontal health should be considered in the etiology of periodontal diseases. The possible role of oral SLT habits in the etiology of periodontal disease among these populations has important implications not only for future research but also for design of public health programs as well as for periodontal treatment planning.

Further studies need to be carried out in high-risk populations with improved methodology and incorporating additional parameters such as type of SLT product used, alveolar bone level, and microbial factors to better understand the relationship between oral SLT habits and periodontal diseases. Knowledge generated from such research endeavors can be combined with the data generated from epidemiologic studies regarding factors contributing to the initiation and maintenance of oral SLT habits so as to design public health programs for tackling the burden of SLT habits. SLT-cessation programs in dental settings have been shown to be effective in increasing the proportion of oral SLT users who quit the habit (72, 73). Hence, tobacco-cessation programs in a dental setting should also include smokeless forms. Once such modifications are successfully implemented, the periodontist can play a greater role in tackling this social menace of SLT consumption, thereby contributing to a reduction in the economic burden arising from these habits as well as an improvement of oral and general health and related quality of life.

## Conflict of Interest Statement

The authors declare that the research was conducted in the absence of any commercial or financial relationships that could be construed as a potential conflict of interest.
